# Temporal Trends and Identification of Suicide Mortality Risk Areas in Brazil (2000–2022): Are We Dealing with an Underestimated Epidemic?

**DOI:** 10.3390/medicina60122083

**Published:** 2024-12-19

**Authors:** Danilo de Gois Souza, Lucas Almeida Andrade, José Augusto Passos Góes, Luís Ricardo Santos de Melo, Matheus Santos Melo, Caíque Jordan Nunes Ribeiro, José Marcos de Jesus Santos, Emerson Lucas Silva Camargo, Álvaro Francisco Lopes de Sousa, Liliane Moretti Carneiro, Regina Claudia da Silva Souza, Márcio Bezerra Santos, Shirley Veronica Melo Almeida Lima, Carla Aparecida Arena Ventura, Allan Dantas dos Santos

**Affiliations:** 1 Collective Health Research Center (NISC/UFS), Postgraduate Program in Nursing, Federal University of Sergipe, São Cristóvão 49107-230, SE, Brazil; danilodegoissouza@academico.ufs.br (D.d.G.S.); goesmv@hotmail.com (J.A.P.G.); luisricardo01@academico.ufs.br (L.R.S.d.M.); caiquejordan@academico.ufs.br (C.J.N.R.); shirleylima@academico.ufs.br (S.V.M.A.L.); allanufs@hotmail.com (A.D.d.S.); 2 Collective Health Research Center (NISC/UFS), Postgraduate Program in Health Sciences, University of Sergipe, São Cristóvão 49107-230, SE, Brazil; lucas_almeidandrade@hotmail.com; 3 Postgraduate Program in Tropical Medicine, University of Brasília, Campus Darcy Ribeiro, Asa Norte, Brasília 70910-900, DF, Brazil; matheussmelo@live.com; 4 College of Nursing Collective Health Research Center (NISC/UFS), University Tiradentes, Aracaju 49037-120, SE, Brazil; jsmarcos.ufs@hotmail.com; 5 Ribeirão Preto College of Nursing, Universidade de São Paulo, Ribeirão Preto 14040-902, SP, Brazil; lucmrg0@gmail.com (E.L.S.C.); caaventura@eerp.usp.br (C.A.A.V.); 6 Instituto de Ensino e Pesquisa, Hospital Sírio-Libanês, São Paulo 01308-050, SP, Brazil; regina.souza@hsl.org.br; 7 Program in Health and Development in the Central-West Region, Universidade Federal do Mato Grosso do Sul, Três Lagoas 79603-011, MS, Brazil; liliane-moretti@hotmail.com; 8 Medical and Nursing Science Complex, Federal University of Alagoas, Maceió 57309-005, AL, Brazil; bio_marcio2006@hotmail.com

**Keywords:** suicide, mortality, Brazil, spatial distribution, temporal trends

## Abstract

*Background and Objectives*: Suicide is a pressing public health issue globally, including in Brazil, where it ranks among the leading causes of mortality. This study aimed to analyze the spatial, temporal, and spatiotemporal distribution of suicide mortality in Brazil from 2000 to 2022. *Materials and Methods*: Using secondary data from the Mortality Information System of Brazil’s 5570 municipalities, an ecological study of time series was conducted. Segmented linear regression (Joinpoint 4.6 version) was used to calculate temporal trends, while Moran’s indices were employed to analyze spatial autocorrelations. Retrospective scanning was utilized to investigate spatiotemporal clusters, and choropleth maps were developed to visualize high-risk areas. *Results*: The analysis revealed the occurrence of 240,843 suicides in Brazil, with higher percentages in the southeast, south, and northeast regions. The south, central–west, and southeast regions exhibited the highest mortality rates, predominantly among white, single men, aged 20 to 59, with 1 to 11 years of schooling. Intentional self-harm by hanging, strangulation, and suffocation was the main cause. The general trend of mortality due to suicide in Brazil was increasing (AAPC: 2.9; CI 95%: 2.6 to 3.0), with emphasis on the age groups from 10 to 19 years (AAPC: 3.7; CI 95%: 2.9 to 4.5) and 20–39 years old (AAPC: 2.9; CI 95%: 2.3 to 3.5). The brutal and smoothed rates revealed areas of high mortality in the south, north, and central–west regions. *Conclusions*: The findings of this study highlight the need to direct resources and efforts to the south and midwest regions of Brazil, where suicide rates are the highest. Additionally, implementing targeted prevention programs for young men, who are the most affected, is essential to reduce suicide mortality in these areas.

## 1. Introduction

Suicide is a complex and multifactorial phenomenon that poses a significant challenge for healthcare systems worldwide due to its profound individual, familial, community, and social repercussions. While primarily associated with mental health, its nature extends beyond, involving sociocultural and economic determinants. Analyzing these factors is crucial for identifying individual suicide-related risks [1]. The most common forms of suicide deaths include exogenous poisoning, hanging, and firearm use, influenced by previous mental disorders, sociodemographic factors, psychological aspects, pain, and chronic comorbidities [2,3,4].

The magnitude of the problem is alarming, with over 700,000 lives lost annually, making suicide the fourth leading cause of death among young people aged 15 to 29, more prevalent in low- and middle-income countries. Suicide attempts are estimated to increase dramatically in the future [5,6].

Suicide prevention is a global priority, with goals established by the WHO aiming to reduce the suicide mortality rate by one third by 2030. Despite a global decline in suicide rates between 2010 and 2016, the Americas experienced a worrying increase during that period [7,8].

In Brazil, the Ministry of Health reported an alarming 43% increase in the annual number of suicide deaths between 2000 and 2019, resulting in a mortality rate of 6.6 deaths per 100,000 inhabitants. Despite studies on suicide trends and spatial clusters, no specific analyses of federal units using temporal, spatial, and spatiotemporal techniques were found [9].

Understanding the dynamics of suicide mortality among the federative units of the country is crucial for identifying areas with higher mortality rates. This enables the prioritization of investments for planning and implementing targeted preventive interventions, tailored to the specific characteristics and needs of each state. Additionally, this approach is essential for developing programs and public policies that reflect the health realities of each federative unit, ensuring they are relevant and effective for the local situation [10,11].

The study aimed to analyze the spatial, temporal, and spatiotemporal distribution of suicide mortality in Brazil between 2000 and 2022, providing valuable insights to address this serious public health issue.

## 2. Materials and Methods

### 2.1. Study Type and Design

We conducted a population-based time-series study employing spatial analysis techniques with secondary data, including all suicide deaths that occurred in Brazil between 2000 and 2022. This delimitation was established considering that, starting in 1999, Brazil adopted a new version of the Mortality Information System (SIM), which introduced a new Death Certificate (DC). This update resulted in improvements in data recording, with more detailed completion of the DC. Furthermore, it is important to note that, at the time of our data collection, information on suicide deaths was only available up to the year 2022. The units of analysis were the 5 regions of the country, its 27 federative units (UFs), and 5570 municipalities.

### 2.2. Study Area

Brazil is located in South America, being the largest country on this continent, with an area of 8,515,767.049 km^2^. The Brazilian population is approximately 210 million inhabitants, ranking fifth in the world. The country is politically and administratively divided into 27 UFs (26 states and 1 federal district), with its capital in Brasília. For political and operational purposes, the federative units are grouped into five regions (north, northeast, southeast, south, and midwest), each with distinct geographical and cultural characteristics and significant socioeconomic disparities [12].

### 2.3. Data Source

The data on suicide deaths were collected from the SIM of the Brazilian Ministry of Health, publicly available on the website of the Department of Informatics of the Unified Health System (DATASUS). The SIM plays a fundamental role in the process of collecting, storing, and managing death records in the country, using the DC as a standard document, which consists of a form filled out by medical professionals for all deaths occurring in Brazil. For this collection, the codes of the International Classification of Diseases 10th Revision (ICD-10) were used: X60 to X84. Population data were obtained from the Brazilian Institute of Geography and Statistics (IBGE), based on population censuses conducted in 2000, 2010, and 2022, as well as estimates for intercensal years (2001–2009 and 2011–2021). Additionally, the digital cartographic mesh of Brazil, divided by states and regions, was extracted by the Geographic Projection System, in shapefile format (Geodetic Reference System, SIRGAS/2000), also provided by the IBGE [12].

### 2.4. Variables and Measures

The variables used were the number of suicide deaths recorded in the 5570 municipalities of Brazil, considering the entire Brazilian territory, regions, states, and municipalities, and the resident population and the constant proportion of 100,000 inhabitants. Epidemiological data such as age group, gender, marital status, race/skin color, schooling, and place of occurrence and cause of death were used.

### 2.5. Analysis of Epidemiological Data

A descriptive analysis of the epidemiological and demographic characteristics of suicide deaths in Brazil between 2000 and 2022 was conducted. The data were analyzed using Microsoft Office Excel 2016 software and presented in tables with absolute and relative values. Suicide mortality rates were calculated considering the number of deaths per year as the numerator and the resident population as the denominator, expressed per 100,000 inhabitants. To minimize the instability caused by the random fluctuation of deaths, the Local Empirical Bayesian estimator was applied, smoothing the rates through weighted averages and generating a second corrected coefficient. The Empirical Bayesian Rate was used to correct the multiplicative rate to 100,000, taking into account the population at risk and the number of cases for each year, aiming to reduce random fluctuations and improve the visualization of gradients on a large scale. Categorized variables, such as gender, race/color, age group, years of schooling, and cause of death according to the ICD-10 code, were described for Brazil and its regions through absolute and relative frequencies.

### 2.6. Time Trends Analysis

An analysis was conducted using the Poisson regression method using Joinpoint Regression™ 4.6 software. This statistical technique allows us to examine changes in the trend of an indicator over time by adjusting the data of a series with the fewest possible inflection points. The statistical significance test for choosing the best model was based on the Monte Carlo permutation method, considering a significance level of *p* < 0.05. Annual percent change (APC) was estimated and tested for each straight segment, and the average annual percent change (AAPC) was tested to quantify the trend over the entire time interval, with 95% confidence intervals (CI 95%). A positive and significant APC indicates an increasing trend, while a negative and significant APC indicates a decreasing trend. Non-significant trends were described as stable, regardless of the APC values. The final selected model was the most adjusted, allowing the best representation of the trend, with the fewest inflection points [13].

### 2.7. Spatial and Space–Time Analysis

To assess the presence of spatial autocorrelation in the mortality coefficient, we calculated the global Moran’s index (I), which gauges the correlation of the variable with itself across space. A spatial proximity matrix was established using the contiguity criterion, with a significance level of 5%. The global Moran’s index ranges from −1 to +1, where values near zero signify spatial randomness [14].

After identifying spatial autocorrelation, we examined local autocorrelation by computing the local Moran’s index (LISA), which reveals the dependence of local data on neighboring areas and facilitates the detection of spatial association patterns. These analyses were conducted using TerraView 4.2.2, and choropleth maps were generated in QGIS 3.4.11 [15].

We conducted a space–time analysis to pinpoint clusters of high suicide mortality risk in Brazil. Retrospective space–time scanning statistics were employed, utilizing the Poisson distribution probability model with specific parameters [16]. The primary and secondary clusters were identified via the log-likelihood ratio test (LLR) and illustrated through maps and tables. These analyses were executed using SaTScan™ 9.6 software [17].

### 2.8. Ethical Considerations

This study utilized aggregated secondary data from the public domain, thus necessitating no submission to a research ethics committee. We adhered to the ethical guidelines outlined in Resolution 466/2012 of the National Health Council (CNS) and the Helsinki Declaration.

## 3. Results

A total of 240,843 suicide deaths were recorded in Brazil. The highest percentages of cases were distributed in the southeast (37.59%), south (23.72%), and northeast (22.64%) regions of the country. The majority of death cases corresponded to males (78.74%), white individuals (51.18%), unmarried individuals (50.26%), adults aged 20 to 59 years (75.35%) with 1 to 11 years of schooling (57.87%). Regarding the location of occurrence, the research also indicated that over half of the deaths (59.16%) occurred in residences (Table 1).

Among the causes of suicide deaths in Brazil, we identified that the majority were caused by intentionally self-inflicted injury by hanging, strangulation, and suffocation—ICD X70 (64.13%); followed by intentional self-harm by discharge of other firearms and unspecified firearms—ICD X74 (7.27%); and intentional self-poisoning and exposure to pesticides—ICD X68 (5.04%). Together, these causes accounted for over 76% of all suicide deaths in Brazil between the years 2000 and 2022 (Table 2).

The suicide mortality rates in Brazil varied from 3.91 (2000) to 8.11 deaths (2022) per 100,000 inhabitants. The average rate in the country for the period was 5.30 deaths per 100,000 inhabitants. The highest coefficients were found in the south (12.35/100,000 inhabitants) and midwest (9.79/100,000 inhabitants) regions (Supplementary Materials, Table S1).

The temporal analysis of suicide mortality in Brazil by region, sex, and age group revealed an increasing trend (AAPC: 2.9; CI 95%: 2.6 to 3.0) for Brazil in the years studied. Increasing trends were also identified in all five regions of the country, with an emphasis on the northeast (AAPC: 4.3; CI 95%: 3.9 to 5.0) and north (AAPC: 4.1; CI 95%: 3.5 to 4.7) regions, thus surpassing the national trend (Table 3).

Furthermore, it was observed that both sexes exhibited an increasing trend in mortality, albeit with slight differences in the percentage of variation between females (AAPC: 2.9; CI 95%: 2.6 to 3.3) and males (AAPC: 3.0; CI 95%: 2.7 to 3.2). With the exception of the population aged 0 to 9 years, which maintained a stable trend, all other age groups showed increased rates, particularly the age groups of 10 to 19 years (AAPC: 3.7; CI 95%: 2.9 to 4.5) and 20 to 39 years (AAPC: 2.9; CI 95%: 2.3 to 3.5) (Table 3).

**Figure 1:**
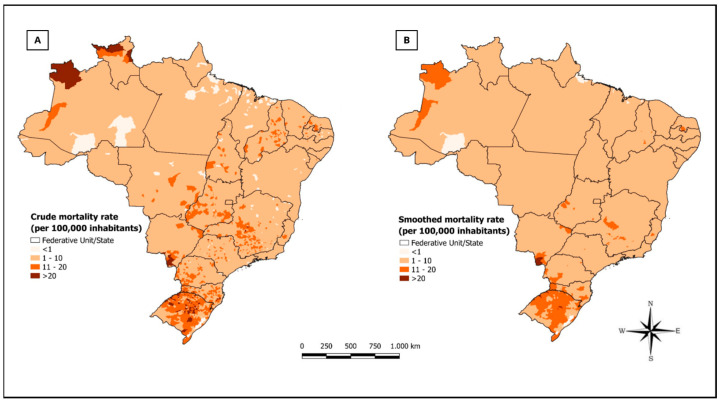
Spatial analysis of suicide mortality in the regions of Brazil, 2000 to 2022. (**A**) Crude mortality rate. (**B**) Smoothed mortality rate.

The distribution of crude mortality rates and smoothed suicide rates is displayed in the maps represented in Figure 1A,B, respectively. It was observed that the crude rates reveal areas of high mortality in the south, midwest, and north of the country. As for the smoothed rates, they exhibit higher indices in the south and north regions, with more discreet numbers in the midwest region.

**Figure 2:**
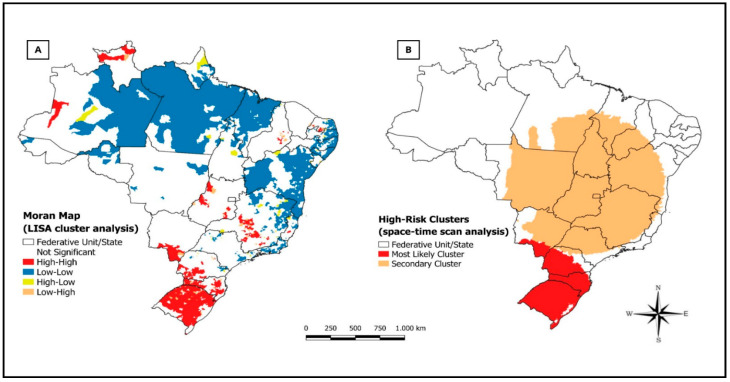
Spatial analysis of suicide mortality in the regions of Brazil, 2000 to 2022. (**A**) Moran map (LISA cluster). (**B**) Spatiotemporal scanning analysis.

The analysis of spatial autocorrelation was obtained by calculating the global Moran’s index (Figure 2A), which revealed a statistically significant result, indicating the existence of spatial dependence among deaths in states with similar patterns. The Moran map demonstrates clusters of states identified in the scatterplot obtained by univariate LISA.

Space–time analysis was conducted through space–time scanning statistics. Two statistically significant space–time clusters of suicide mortality risk were identified, visualized in Figure 2B. The primary cluster presents the highest risk for suicide deaths, comprising the states of Mato Grosso do Sul (MS), Paraná (PR), Santa Catarina (SC), and Rio Grande do Sul (RS), between the years 2012 and 2022, corresponding to 1055 municipalities (RR = 2.15; *p* < 0.001) (Supplementary Material Table S2).

## 4. Discussion

Brazil has witnessed a significant increase in suicide mortality rates, with a growing trend throughout its territory, especially in the southeast, south, and northeast regions [18]. As the largest country in Latin America in terms of territorial extension, Brazil exhibits diverse social, cultural, and economic characteristics, reflected in the disparate patterns in suicide incidence and mortality. A study covering the period from 1996 to 2019 observed an increase in the mortality rate, rising from 4.3 to 6.4 per 100,000 inhabitants, representing a 48.8% increase. While most regions of the world experienced a reduction in suicide rates, the southern and central–western regions of Brazil stand out with the highest rates [19].

Suicide mortality rates reveal striking disparities between genders, with men showing a much higher prevalence compared to women. These discrepancies may be associated with various social realities experienced by each individual. Men are often perceived as the primary providers for the family, increasing the chances of stressful events related to work and finances. Additionally, typical masculine behaviors such as competitiveness, impulsivity, and easy access to firearms may predispose men to the risk of suicide. It is crucial to highlight that men tend to pay less attention to their mental health, which can have adverse consequences for their physical, social, and psychological well-being [10,20].

Adolescents and young adults represent a significant portion of suicide deaths, and countries like Japan, the United States, Portugal, and Ecuador have also observed an increase in suicide rates in this age group [21]. The economic crisis is one of the factors associated with this increase, especially affecting young people trying to establish themselves in the job market. This group faces a variety of stressful events, such as career choice, family independence, relationships, transition to university life, and access to psychoactive substances, all contributing to a higher risk of suicide [21].

Educational level plays a crucial role in suicide mortality, with a higher incidence among individuals with advanced educational backgrounds due to the pressure associated with high expectations and losses in social status during economic crises. However, low educational attainment also presents worrying risks, increasing unemployment and triggering economic problems and feelings of guilt and hopelessness, predisposing to suicide [22].

The majority of suicide cases occurred in the victim’s residential environment, possibly due to the use of more discreet methods and the privacy offered, making practical interventions difficult and requiring effective prevention strategies [23].

Temporal analysis of suicide mortality in Brazil revealed a consistent and significant increase across the territory, especially in the north and northeast regions, attributed to socioeconomic changes, financial instability, unemployment, and inadequate treatment of mental disorders. Both genders showed increasing trends over time, highlighting the urgent need for new suicide prevention strategies [23,24,25].

The significant and consistent increase in suicide deaths among adolescents and young adults is a growing concern, as evidenced by previous studies that have identified high mortality rates in this age group. Factors such as lower religious support, family cohesion, unemployment, and substance abuse contribute to this trend. This underscores the urgency of implementing health strategies to reduce suicide risks in this population [26].

Mapping clusters of high mortality risk revealed a primary cluster covering the southern and central–western states of Brazil, where sociocultural, economic, and psychobiological factors among farmers are related to high suicide rates. The predominant cultivation of tobacco, pesticide use, mental disorders, and behavioral patterns stemming from colonization by European immigrants are key elements in this scenario [27].

These findings highlight the relevance of the issue, revealing the temporal evolution of suicide mortality and its geographical distribution as a serious public health problem. They provide a deeper understanding of the impact of suicide in different regions of the country, promoting the formulation of public policies aimed at suicide prevention at both the national and regional levels [27,28].

This study offers significant contributions to mental health nursing. By analyzing the temporal and spatial trends of suicide mortality in Brazil, it provides crucial insights for nurses and other mental health professionals to understand the complexity of this problem at both the national and regional levels. This understanding is essential to inform the development of nursing interventions tailored to the specific needs of different demographic groups and locations.

In this context, suicide prevention requires the implementation of public policies and comprehensive, integrated actions involving various sectors of society. Key initiatives include improving and expanding the support network, training healthcare professionals to assess and monitor at-risk individuals, conducting educational and awareness campaigns with a special focus on vulnerable groups such as adolescents and young adults, and establishing mental health support programs in collective environments like schools and universities [9].

Furthermore, by identifying areas of higher risk and vulnerable population groups, the study empowers nurses to direct resources and intervention efforts where they are most needed, thereby contributing to suicide prevention and mental health promotion across the Brazilian population. These findings provide a solid foundation for the implementation of effective and targeted nursing strategies aimed at reducing the impact of suicide and improving community mental well-being.

To better understand the rising trend in suicide deaths in Brazil and the observed regional variations, further studies are needed to investigate how factors such as socioeconomic determinants, digital and social media, and the COVID-19 pandemic may influence suicide rates in the country.

### 4.1. Limitations

This study acknowledges significant limitations in its approach. One of them is the nature of the secondary data used, which comes from a system subject to inconsistencies in quality due to incorrect information filling. This variability can affect the results, requiring caution in interpretation.

Additionally, there is recognition of the possibility of underreporting due to inappropriate classifications of causes of death, which may underestimate the true incidence of suicides. The study, being ecological, may not precisely capture the individual experiences of the subjects.

### 4.2. Relevance for Clinical Practice

Understanding the temporal and spatial trends of suicide mortality in Brazil provides valuable insights for healthcare professionals, enabling them to adapt interventions and services to meet the specific needs of at-risk populations. Healthcare providers, including nurses, psychiatrists, psychologists, and social workers, can utilize this information to enhance their assessment, diagnosis, and treatment of individuals at risk of suicide. By recognizing demographic groups and geographic areas with higher suicide rates, clinicians can implement targeted interventions and preventive measures to address underlying risk factors and promote mental well-being.

Moreover, identifying groups with a high risk of suicide enables healthcare professionals to allocate resources effectively and implement community interventions aimed at reducing suicide rates in these areas. Collaborative efforts between healthcare providers, community organizations, and policymakers are essential to develop and implement comprehensive suicide prevention strategies that address the multifaceted nature of this public health issue.

## 5. Conclusions

Suicide mortality emerges as a pressing public health challenge in Brazil, with a growing trend observed in all regions of the country, especially in the northeast and north. There is a significant increase among men, adolescents, and young adults. This issue reveals a heterogeneous distribution, with the southern and central–western regions identified as areas of higher risk.

Faced with this alarming panorama, it becomes imperative to invest in comprehensive and intersectoral public policies throughout the national territory. These policies should be adapted to the specificities of each region, aiming for an effective allocation of resources and a strategic focus on suicide prevention and control. Prioritizing the most affected areas is essential to substantially reduce mortality rates, thus promoting the mental health and well-being of the Brazilian population.

## Figures and Tables

**Table 1:** Sociodemographic characteristics of suicide mortality in Brazil and its regions, 2000 to 2022.

Variables	North	Northeast	Southeast	South	Central–West	Brazil
	n (%)	n (%)	n (%)	n (%)	n (%)	n (%)
Sex						
Male	13,015 (78.56)	43,188 (79.18)	70,642 (78.02)	45,667 (79.92)	17,129 (77.69)	189,641 (78.74)
Female	3546 (21.41)	11,345 (20.80	19,888 (21.96)	11,467 (20.07	4916 (22.30)	51,162 (21.24)
Unknown	5 (0.03)	12 (0.02)	15 (0.02)	4 (0.01)	4 (0.02)	40 (0.02)
Race/Ethnicity						
White	2411 (14.55)	8784 (16.10)	54,577 (60.28)	49,626 (86.85)	7863 (35.66)	123,261 (51.18)
Non-white	13,802 (83.32)	41,414 (75.93	33,396 (36.88)	6696 (11.72)	13,641 (61.87)	108,949 (45.24)
Unknown	353 (2.13)	4347 (7.97)	2572 (2.84)	816 (1.43)	545 (2.47)	8633 (3.58)
Age Group						
≤9	33 (0.20)	36 (0.07)	17 (0.02)	12 (0.02)	7 (0.03)	105 (0.04)
10 to 19	2984 (18.01)	4935 (9.05)	5771 (6.37)	3675 (6.43)	2503 (11.35)	19,868 (8.25)
20 to 39	8443 (50.97)	23,999 (44.00)	39,663 (43.80)	20,560 (35.98)	10,156 (46.06)	102,821 (42.69)
40 to 59	3571 (21.56)	16,706 (30.63)	31,283 (34.55)	20,701 (36.23)	6392 (28.99)	78,653 (32.66)
≥60	1485 (8.96)	8782 (16.10)	13,474 (14.88)	12,134 (21.24)	2928 (13.28)	38,803 (16.11)
Unknown	50 (0.30)	87 (0.16)	337 (0.37)	56 (0.10)	63 (0.29)	593 (0.25)
Educational Level						
None	1247 (7.53)	5945 (10.90)	1826 (2.02)	1612 (2.82)	1087 (4.93)	11,717 (4.86)
1 to 3 years	2721 (16.43)	10,824 (19.84)	8805 (9.72)	8538 (14.94)	2829 (12.83)	33,717 (14.00)
4 to 7 years	4624 (27.91)	11,339 (20.79)	19,511 (21.55)	14,128 (24.73)	5040 (22.86)	54,642 (22.69)
8 to 11 years	4318 (26.07)	8419 (15.43)	21,538 (23.78)	11,890 (20.81)	4857 (22.03)	51,022 (21.18)
≥12 years	1146 (6.92)	3005 (5.51)	9069 (10.02)	4182 (7.32)	2217 (10.05)	19,619 (8.15)
Unknown	2510 (15.15)	15,011 (27.52)	29,796 (32.91)	16,788 (29.38)	6019 (27.30)	70,124 (29.12)
Place of Occurrence						
Hospital or other health establishment	2220 (13.40)	9756 (17.89)	20,821 (23.00)	7671 (13.43)	4059 (18.41)	44,527 (18.49)
Residence	10,961(66.17)	32,023 (58.71)	49,387 (54.54)	37,107 (64.94)	13,009 (59.00)	142,487 (59.16)
Public road	722 (4.36)	3904 (7.16)	5780 (6.38)	3229 (5.65)	1210 (5.49)	14,845 (6.16)
Others	2544 (15.36)	8337 (15.28)	13,957 (15.41)	8808 (15.42)	3665 (16.62)	37,311 (15.49)
Unknown	119 (0.72)	525 (0.96)	600 (0.66)	323 (0.57)	106 (0.48)	1673 (0.69)
Marital Status						
Single	10,730 (64.77)	29,629 (54.32)	44,736 (49.41)	24,328 (42.58)	11,624 (52.72)	121,047 (50.26)
Married	2699 (16.29)	14,374 (26.35)	25,773 (28.46)	19,702 (34.48)	5379 (24.40)	67,927 (28.20)
Others	1986 (11.99)	5824 (10.68)	13,105 (14.47)	91,90 (16.08)	3398 (15.41)	33,503 (13.91)
Unknown	1151 (6.95)	4718 (8.65)	6931 (7.65)	3918 (6.86)	1648 (7.47)	18,366 (7.63)
**Total**	**16,566 (6.87)**	**54,545 (22.64)**	**90,545 (37.59)**	**57,138 (23.72)**	**22,049 (9.15)**	**240,843 (100)**

**Table 2:** Leading causes of suicide deaths in Brazil and its regions, 2000 to 2022.

ICD-10 Codes	North	Northeast	Southeast	South	Central–West	Brazil
	n (%)	n (%)	n (%)	n (%)	n (%)	n (%)
X60	24 (0.14)	34 (0.06)	78 (0.09)	76 (0.13)	14 (0.06)	226 (0.09)
X61	78 (0.47)	533 (0.98)	1858 (2.05)	953 (1.67)	475 (2.15)	3897 (1.62)
X62	80 (0.48)	193 (0.35)	488 (0.54)	269 (0.47)	129 (0.59)	1159 (0.48)
X63	7 (0.04)	56 (0.10)	90 (0.10)	51 (0.09)	33 (0.15)	237 (0.1)
X64	270 (1.63)	1513 (2.77)	1996 (2.20)	1121 (1.96)	488 (2.21)	5388 (2.24)
X65	79 (0.48)	305 (0.56)	277 (0.31)	151 (0.26)	80 (0.36)	892 (0.37)
X66	28 (0.17)	63 (0.12)	105 (0.12)	49 (0.09)	20 (0.09)	265 (0.11)
X67	15 (0.09)	39 (0.07)	283 (0.31)	151 (0.26)	59 (0.27)	547 (0.23)
X68	806 (4.87)	4303 (7.89)	3992 (4.41)	1648 (2.88)	1401 (6.35)	12,150 (5.04)
X69	348 (2.10)	2198 (4.03)	2713 (3.00)	766 (1.34)	631 (2.86)	6656 (2.76)
X70	12,321 (74.38)	35,643 (65.35)	53,149 (58.70)	39,490 (69.11)	13,850 (62.81)	154,453 (64.13)
X71	136 (0.82)	503 (0.92)	1086 (1.20)	818 (1.43)	181 (0.82)	2724 (1.13)
X72	525 (3.17)	1147 (2.10)	1707 (1.89)	2413 (4.22)	768 (3.48)	6560 (2.72)
X73	166 (1.00)	390 (0.72)	134 (0.15)	261 (0.46)	134 (0.61)	1085 (0.45)
X74	864 (5.22)	2730 (5.01)	6716 (7.42)	5324 (9.32)	1875 (8.5)	17,509 (7.27)
X75	4 (0.02)	25 (0.05)	53 (0.06)	14 (0.02)	11 (0.05)	107 (0.04)
X76	91 (0.55)	919 (1.68)	2162 (2.39)	544 (0.95)	304 (1.38)	4020 (1.67)
X77	5 (0.03)	20 (0.04)	54 (0.06)	17 (0.03)	9 (0.04)	105 (0.04)
X78	273 (1.65)	701 (1.29)	1653 (1.83)	826 (1.45)	436 (1.98)	3889 (1.61)
X79	76 (0.46)	463 (0.85)	1075 (1.19)	103 (0.18)	96 (0.44)	1813 (0.75)
X80	187 (1.13)	1350 (2.48)	5120 (5.65)	1348 (2.36)	655 (2.97)	8660 (3.6)
X81	12 (0.07)	58 (0.11)	285 (0.31)	77 (0.13)	30 (0.14)	462 (0.19)
X82	21 (0.13)	148 (0.27)	677 (0.75)	132 (0.23)	95 (0.43)	1073 (0.45)
X83	21 (0.13)	159 (0.29)	213 (0.24)	86 (0.15)	48 (0.22)	527 (0.22)
X84	129 (0.78)	1052 (1.93)	4581 (5.06)	450 (0.79)	227 (1.03)	6439 (2.67)
**Total**	**16,566**	**54,545**	**90,545**	**57,138**	**22,049**	**240,483**

X60: Intentional self-poisoning and exposure to non-opioid analgesics, antipyretics, and anti-rheumatics. X61: Intentional self-poisoning and exposure to anticonvulsants, sedatives, hypnotics, antiparkinsonian drugs, and psychotropic drugs. X62: Intentional self-poisoning and exposure to narcotics and psychodysleptics [hallucinogens] not classified elsewhere. X63: Intentional self-poisoning and exposure to other pharmacological substances affecting the autonomic nervous system. X64: Intentional self-poisoning and exposure to other drugs, medications, and unspecified biological substances. X65: Voluntary self-poisoning with alcohol. X66: Intentional self-poisoning with organic solvents, halogenated hydrocarbons, and their vapors. X67: Intentional self-poisoning with other gases and vapors. X68: Intentional self-poisoning and exposure to pesticides. X69: Intentional self-poisoning and exposure to other chemicals and unspecified harmful substances. X70: Intentional self-harm by hanging, strangulation, and suffocation. X71: Intentional self-harm by drowning and submersion. X72: Intentional self-harm by manually firing a firearm. X73: Intentional self-harm by firing a shotgun or firearm of larger caliber. X74: Intentional self-harm by firing another firearm and an unspecified firearm. X75: Intentional self-harm by explosive devices. X76: Intentional self-harm by smoke, fire, and flames. X77: Intentional self-harm by steam, gases, or hot objects. X78: Intentional self-harm by sharp or penetrating object. X79: Intentional self-harm by blunt object. X80: Intentional self-harm by jumping from a high place. X81: Intentional self-harm by jumping or standing in front of a moving object. X82: Intentional self-harm by impact of a motor vehicle. X83: Intentional self-harm by other specified means. X84: Intentional self-harm by unspecified means. Source: Authors.

**Table 3:** Temporal trend of suicide mortality rates by region, sex, and age group in Brazil, 2000–2022.

Variables	Period	APC (CI 95%)	Trend	AAPC (CI 95%)	Trend
Country/Region					
Brazil	2000–2015	1.7 * (0.75 to 2.1)	Increasing	2.9 * (2.6 to 3.0)	Increasing
	2015–2020	3.9 * (3.6 to 6.5)	Increasing		
	2020–2022	9.4 * (5.8 to 11.7)	Increasing		
North	2000–2017	3.2 * (1.1 to 3.9)	Increasing	4.1 * (3.5 to 4.7)	Increasing
	2017–2022	7.3 * (4.6 to 14.0)	Increasing		
Northeast	2000–2006	6.9 * (4.7 to 12.4)	Increasing	4.3 * (3.9 to 5.0)	Increasing
	2006–2015	1.3 (−3.5 to 2.5)	Stable		
	2015–2022	6.1 * (4.6 to 9.9)	Increasing		
Southeast	2000–2020	2.1 * (1.7 to 2.3)	Increasing	2.8 * (2.5 to 3.1)	Increasing
	2020–2022	10.9 * (5.0 to 14.1)	Increasing		
South	2000–2014	0.0 (−0.4 to 0.4)	Stable	1.7 * (1.5 to 2.0)	Increasing
	2014–2022	4.9 * (4.1 to 6.1)	Increasing		
Central–West	2000–2015	−0.7 (−0.8 to0.7)	Stable	1.9 * (1.5 to 2.4)	Increasing
	2015–2022	6.1 * (4.4 to 9.4)	Increasing		
Sex					
Male	2000–2015	1.7 * (0.6 to 2.1)	Increasing	3.0 * (2.7 to 3.2)	Increasing
	2015–2020	3.7 * (1.8 to 5.3)	Increasing		
	2020–2022	10.9 * (7.0 to 13.4)	Increasing		
Female	2000–2016	1.8 * (1.1 to 2.3)	Increasing	2.9 * (2.6 to 3.3)	Increasing
	2016–2022	6.0 * (4.5 to 9.6)	Increasing		
Age Group					
0–9	2000–2022	0.7 (−2.3 to 3.7)	Stable	0.7 (−2.3 to 3.7)	Stable
10–19	2000–2013	0.5 (−0.5 to 1.5)	Stable	3.7 * (2.9 to 4.5)	Increasing
	2013–2022	8.5 * (7.0 to 10.0)	Increasing		
20–39	2000–2016	1.3 * (1.0 to 1.5)	Increasing	2.9 * (2.3 to 3.5)	Increasing
	2016–2020	3.8 * (1.3 to 6.4)	Increasing		
	2020–2022	15.5 * (10.5 to 20.8)	Increasing		
40–59	2000–2012	0.7 * (0.1 to 1.2)	Increasing	1.9 * (1.2 to 2.6)	Increasing
	2012–2020	2.0 * (1.0 to 3.1)	Increasing		
	2020–2022	8.5 * (1.7 to 15.8)	Increasing		
≥60	2000–2014	0.7 * (0.2 to 1.1)	Increasing	1.1 * (0.7 to 1.5)	Increasing
	2014–2022	1.9 * (1.1 to 2.8)	Increasing		

* *p*-value < 0.05; APC: annual percentage changes; AAPC: average annual percentage changes; IC: confidence interval.

## Data Availability

All data generated and analyzed during this study are publicly available from the Brazilian Ministry of Health’s Department of Informatics (DATASUS) and the Brazilian Institute of Geography and Statistics (IBGE) websites.

## Supplementary Materials

The following supporting information can be downloaded at https://www.mdpi.com/article/10.3390/medicina60122083/s1, Table S1. Suicide mortality rates in Brazil and regions, 2000 to 2022. Table S2. Spatio-temporal clusters of suicide mortality in Brazil, 2000 to 2022.
