# Supplementary material for: Temporal Trends and Identification of Suicide Mortality Risk Areas in Brazil (2000–2022): Are We Dealing with an Underestimated Epidemic?
Source: Medicina (Kaunas). 2024 Dec 19;60(12):2083. doi: 10.3390/medicina60122083 (PMC11677106; doi:10.3390/medicina60122083)
Supplement: Supplementary file 1 [file medicina-60-02083-s001.zip › medicina-3125494-supplementary.pdf]

Supplementary material 1 - Suicide mortality rates in Brazil and regions, 2000 to 2022.

	Initial	Final	Average	Minimum	Maximum
BRAZIL	3.91	8.11	5.30	3.91	8.11
North	2.80	7.56	4.32	2.80	7.56
Northeast	2.31	7.02	4.32	2.31	7.02
Southeast	3.40	7.10	4.73	3.40	7.10
South	8.01	12.35	8.77	7.61	12.35
Central-West	6.13	9.79	6.56	5.50	9.79

Supplementary material 2 - Spatio-temporal clusters of suicide mortality in Brazil, 2000 to 2022.

Cluster	Time period	Number of municipalities	States	Observed	Expected	RR	LLR	p-value
Period								
1	2012 - 2022	1,055	MS, PR, SC, RS	26,482	13,090	2,15	5,670.31	<0.001
2	2012 - 2022	2,376	PA, TO, MA, PI, BA, MT, MS, GO, DF, MG, ES, SP, PR	30,376	20,999	1,51	2,040.55	<0.001

Notes:

- RR: Relative Risk
- LLR: Log-Likelihood Ratio
- p-value: Probability value
- MS: Mato Grosso do Sul
- PR: Paraná
- SC: Santa Catarina
- RS: Rio Grande do Sul
- PA: Pará
- TO: Tocantins
- MA: Maranhão
- PI: Piauí
- BA: Bahia
- MT: Mato Grosso
- GO: Goiás
- DF: Federal District
- MG: Minas Gerais
- ES: Espírito Santo
- SP: São Paulo
